# Consumer knowledge and availability of maternal and child health services: a challenge for achieving MDG 4 and 5 in Southeast Nigeria

**DOI:** 10.1186/1472-6963-13-53

**Published:** 2013-02-09

**Authors:** Nwala K Emmanuel, Ebunoha N Gladys, Ugwu U Cosmas

**Affiliations:** 1 Department of Health and Physical Education, University of Nigeria, Nsukka, Nigeria; 2 Children Out-Patient Clinic, Enugu State University Teaching Hospital [ESUTH], Parklane, Enugu, Nigeria; 3 Health Policy Research Group, Department of Pharmacology and Therapeutics, College of Medicine, UNEC, Enugu, Nigeria

## Abstract

**Background:**

Reducing child mortality and improving maternal health occupies a prominent space in the Millennium Development Goals (MDGs), and it has been noted that some reductions have taken place, but not enough. If consumers know what and where services are available, they may be motivated to use them. This study therefore evaluated consumers’ knowledge about available maternal and child health services and where these services can be obtained in the study area. Although knowledge of available health services does not translate to utilization of these services, this study is important as knowledge of available health services can prompt the informed use of services. The study determined the consumers’ knowledge about available Maternal and Child Health services and where these services are available.

**Methods:**

The study was a cross-sectional research design. The sample for the study consisted of a total of 450 women of child bearing age selected from the 20 political wards that make up Ezeagu Local Government Area. The 20 political wards constituted 20 clusters (cluster sampling technique) i.e. one cluster per political ward. Simple random sampling method by balloting was used to select five (5) wards out of the 20 political wards. Finally, a total of 90 women of childbearing age were selected from each of the five wards (clusters) using simple random method.

**Results:**

The study showed that majority of the women (37.3%) were between 36-45 years, married [49.5%], had more than five children [21.6%], hold at least SSCE [23.7%], and were farmers and Christians [32.3% and 81.8%] respectively. Maternal health services available are mainly antenatal [57%] and delivery services [54.3%]. Other available services are described at the results section. In the same vein, immunization [63.8%] was the most available child health service in the area. Both Maternal and Child Health services were available mainly at public and private hospitals [53.6% and 52.3% for maternal services; 56.1% and 53.9% respectively for child health services] respectively [see result section for details].

**Conclusions:**

Available Maternal and Child Health services known to mothers in the study area were not encouraging, and these are structurally contextual. ANC and delivery services for mothers, and immunization for children were found to be available as indicated by at least more than half of the respondents. The women knew that these services were available mostly in public and private hospitals which should constitute referral points instead of the health centers that offer primary care at community level. Knowledge of available services is important for consumers to make use of the services. Awareness programmes should be targeted more on the consumers if the MDG 4 and 5 must be reached by 2015**.** This suggests that the women in the study area do not use primary health care services adequately, and may be incurring huge indirect costs and at the same time travel too far to obtain primary care. This is therefore quite challenging for reducing child mortality and improving maternal health in southeast Nigeria. Knowledge of available services is important for consumers to make use of the services. Awareness programmes should be targeted more on the consumers if the MDG 4 and 5 must be reached by 2015**.**

## Background

Reducing child mortality and improving maternal health occupy a prominent space in the Millennium Development Goals (MDGs), and it has been noted that some reductions have taken place, but not enough [1-4]. Maternal and Child Health (MCH) services embrace all the services for mothers throughout the child bearing age, that is 15–49 years of age, and also services for children from conception through adolescence [5]. This includes promotive, preventive, curative and rehabilitative healthcare for mothers and under-five children [6].

MDG Goal 4 strives to reduce child mortality. It is targeted to reduce by three-quarters, between 1990 and 2015 child mortality rate [1]. The main interventions which have made significant contributions to the dramatic fall in child mortality rate and represent child health services in most developing countries are: immunization, oral re-hydration, growth monitoring, breast feeding, family planning, female education and supplementary feeding of pregnant women [7,8]. A report in Nigeria indicates that infant, child, and under-five mortality rates in 2008 were 75, 88, and 157 respectively, although it seems to have reduced when compared to previous years [9].

The MDG 5 which is to improve maternal health targets to reduce by three-quarters, between 1990 and 2015, the maternal mortality ratio [1]. This target is indicated by maternal mortality ratio, proportion of births attended by skilled health personnel, contraceptive prevalence rate, adolescent birth rate, antenatal care coverage, and unmet need for family planning [10]. Reports have indicated that the death toll of mothers remains unacceptably high in South Asia and in sub-Saharan Africa [11]. In Nigeria, estimates ranging from 545 to 1000 maternal deaths per 100,000 live births have been put forward [9] with wide geographical disparity. Nigeria is estimated to contribute 10% of the global estimates of maternal deaths and this translates to about one maternal death every three minutes.

These can drastically be reduced if women know and use available and appropriate services in their communities. Studies have found that the use of services are more strongly correlated to socio-economic and demographic phenomenon [5,12], and also related to the organization of health service delivery systems. This is affected by availability, accessibility, quality, cost, social structure, comprehensiveness of service, and health beliefs [13]. However, evidence in Nigeria and from other countries shows that rural women tend to use less of these services for themselves and their children [9,14].

Strong and indispensible is the role of knowledge in utilization of services. This has also been reported in other studies [12,15]. Some other studies strongly showed that women only know more about ANC and delivery services for maternal health and immunization for children [12,16]. For example, a study in Tanzania evaluated use pattern of maternal health services. The results show that use of ANC was universal compared to other services such as skilled delivery, and this decreased with maternal age [12].

The Nigeria Demographic and Health survey shows that 58% of women received some antenatal care (ANC) from a skilled provider, most commonly from a nurse or midwife (30%) or a doctor (23%). About one-third of births in Nigeria (35%) occurred in health facilities—20% in the public sector and 15% in private sector facilities. However, sixty-two percent of the births occurred at home [9].

This study therefore evaluated consumers’ knowledge about available maternal and child health services and where these services are available in the study area. Although knowledge of available health services does not translate to use of these services, his study is important as knowledge of available health services can prompt informed use of services. Furthermore, providers of services and decision makers can envisage the contributions of the knowledge of available MCH services to reducing child mortality and improving maternal health.

## Objectives

The study determined the consumers’ knowledge about available maternal and child health services and where these services are available.

## Methods

### Study area

Ezeagu Local Government Area was created out from Udi Local Government Area in 1975 and the headquarters is located at Aguobu-Owa. It is situated in the Enugu north senatorial district. The Area shares common boundaries with Uzo uwani Local Government Area in the east, Orji River L.G.A in the south, Udi L.G.A in the west and Ebenebe community in Anambra State in the north. It is about 17 km from the State capital. Most of the people are farmers and petty traders. Christianity is widely practiced in the area.

Ezeagu L.G.A is wholly a rural setting with hilly/stone topography and many hard-to-reach areas. The Local Government Area has a population of 169,718, (84,053 males and 85,665 females). The L.G.A is divided into four development council areas, namely Ezeagu Central, Ezeagu South, Ezeagu North and Ezeagu East. There are 20 political wards and 30 health facilities owned by the government scattered all over 25 communities, all sited for easy reach to the people. Ezeagu L.G.A is wholly a rural setting with hilly/stony topography and many hard-to-reach areas.

These 30 public health facilities include four secondary health facility [cottage Hospitals], while others are health centers, health posts and dispensaries. There are very few private hospitals and less than 10 pharmacies as well, with numerous traditional birth attendants and patent medicine dealers, even though drug itinerancy is widely practiced. The people strongly believe in herbal medicine and consent to utilizing TBA services. However, the roads to the facilities are not in good shape and almost impassable and this poses a great challenge to access to the facilities especially during the rainy seasons. At the public health, user fees are not charged except for informal payments which are not usually documented.

### Study design

The study was a cross-sectional research design using a household survey and the sample for the study consisted of a total of 450 women of child bearing age selected from the 20 political wards that make up Ezeagu Local Government Area. The 20 political wards constitute 20 clusters (cluster sampling technique). Simple random technique by balloting was used to select five (5) wards out of the 20 political wards. Below is the breakdown of sample size calculation:

$$N=\frac{Z\alpha^{2}\;P\;\left(1-P\right)}{D^{2}}$$

N = sample Size

Za^2^ = Significance level at 95% or 1.96

P = Prevalence (50% or 0.5)

D^2^ = Error tolerated at 5%

Therefore

$$\begin{array}{l} N\;=\;\frac{1.96^{2}\;\times \;0.5\;\left(1-0.5\right)}{0.05^{2}}\\ \;=\;\frac{3.816\;\times \;0.5\;\left(0.5\right)}{0.0025}\\ \;=\;\frac{1.9208\;\times \;0.50}{0.0025}\\ \;=0.9604\;=384 \end{array}$$

Therefore N (minimum sample size) = 384

Add 17% for non responses = 15/100 × 384 = 66

Total = 450

Finally a total of 450 women of childbearing age were selected for the study. However, 90 women were selected from each of the five wards (clusters) using simple random technique making a total of 450 women.

The instrument for the data collection was a pretested interviewer administered questionnaire designed for the study. The questions were unprompted allowing responses from the respondents without any bias. The questionnaire was administered in the local language [Igbo], unless where the participant was literate enough and demanding no explanations (Additional file 1). Informed consent of the women was duly obtained before the questionnaire was administered. Only those who consented to the study were included. Women of childbearing age who have used the services either for themselves or for their children were included in the study.

## Results

The study showed that majority of the women (37.3%) were between 36–45 years, married [49.5%], had more than five children [21.6%], hold at least SSCE [23.7%], and were farmers and Christians [32.3% and 81.8%] respectively [see Table 1]. Maternal health services available were mainly antenatal [57%] and delivery services [54.3%]. However, other maternal health services indicated to be available were post natal services [45.9%], family planning [44.1%], health education [42%], and others which the respondents did not specify [see Table 2]. These maternal health services were available mainly at public and private hospitals respectively [53.6% and 52.3%] respectively. Other facilities where the services were available include PHC [43.3%], pharmacy [25.5%], and patent medicine dealer’s shop [38.6%] [see Table 2].

**Table 1:** Socio-demographic characteristics of the respondents

**Age distribution**	**No [%]**	**Marital status**	**No [%]**	**Occupation**	**No [%]**
16-25	71 [16.1]	Single	47 [10.8]	Civil servants	142 [32.3]
26-35	122 [27.7]	Married	218 [49.5]	Traders	99 [18]
36-45	164 [37.3]	Widowed	99 [22.5]	Farmers	99 [18]
46-55	48 [10.9]	Divorced	42 [9.5]	Unemployed	66 [15]
>55	34 [7.7]	Separated	30 [6.8]	Professionals	81 [18.64]
None of above	1 [.2]	None of the above	4 [0.9]	Not valid	4 [0.9]
Total	440 [100]	Total	440 [100]	Total	440 [100]
**Number Of Children**		**Educational Qualifications**		**Religion**	
With 1 child	24 [5.5]	No formal education	28 [6.4]	Traditional	47 [10.7]
With 2 children	66 [15]	FSLC	50 [11.4]	Christianity	360 [81.8]
With 3 children	58 [3.2]	WASC/NECO	104 [23.6]	Islam	10 [2.3]
With 4 children	92 [20.9]	Diploma	60 [13.6]	Not indicated	23 [5.2]
With 5 children	75 [17]	NCE	85 [19.3]	Total	440 [100]
With > 5 children	95 [21.6]	B.Sc	73 [16.6]		
None of the above	30 [6.8]	M. Sc	22 [5]		
Total	440 [100]	PHD	17 [3.9]		
		None of the above	1 [0.2]		
		Total	440 [100]		

**Table 2:** Knowledge of available maternal health services and where they are available

**Available maternal services**	**No [%]**
Ante natal services	251 [57.0]
Delivery services	259 [54.3]
Post natal	202 [45.9]
Family planning	194 [44.1]
Health education	185 [42.0]
Others	88 [19.1]
**Where Maternal services are Available**	
Public hospital	236 [53.6]
Private hospital	230 [52.6]
PHC Centers	215 [48.9]
Pharmacy	90 [20.5]
Patent medicine shop	156 [35.5]
Traditional birth attendants	164 [37.3]

Available data on the available child health services in Ezeagu indicate that 63.4% of the services constitute immunization services. Breastfeeding initiative was also indicated [42.5%]. This was closely followed by growth monitoring services (41.8%) and oral re-hydration therapy (40.0%) [see Table 3]. However, these child health services were available in public hospitals (56.1%). Some of the women reported that it was available at private hospitals (53.9%). About 43.3% indicated PHC centers, 38.8% reported patent medicine shop, 28.0% of the women said it was available at the TBAs, while 25.5% indicated that child health services were available at the pharmacy [see Table 3].

**Table 3:** Knowledge of available child health services and where they are available

**Child health services**	**No [%]**
Oral re-hydration	176 [40.0]
Immunization	278 [63.4]
Growth monitoring	184 [41.8]
Breastfeeding initiatives	187 [42.5]
Others	139 [31.6]
Where the services are Available	No [%]
Public hospital	247 [56.1]
Private hospital	237 [53.9]
PHC Centers	208 [43.3]
Pharmacy	112 [25.5]
Patent medicine shop	170 [38.6]
TBA	123 [28.0]

## Discussion

Most of the women surveyed were aged between 36–45 years and had more than five children. There are a lot of implications to this findings: first, the women of this age bracket have huge experiences with child birth, and so could play roles as ‘significant others’ for younger women seeking MCH services. However, other studies have found that use of MCH services decreases with age [9,12]. Secondly, these women may never have sought for services for their children. This finding indicates that the midwives service scheme in Nigeria should target a house to house enlightenment campaign to improve women’s knowledge on available MCH services.

Available maternal and child health services known to mothers in the study area were not encouraging, and these were structurally contextual. ANC and delivery services for mothers, and immunization for children were found mostly to be available as indicated by at least more than half of the respondents. This finding was not surprising as other studies have found the same [12,15,16]. Although the women knew that post natal, family planning, and health education were available for mothers, and oral rehydration therapy, growth monitoring and breastfeeding initiative were available for children, the number of women who possess the knowledge was not encouraging. This finding suggests scaling up awareness campaigns to empower them so they could use MCH services appropriately when they need them.

However, the women knew that these services were available at public and private hospitals other than TBAs and at home. Although this has a lot of access implications especially cost [including travel and time costs] as these women would have to travel a long way to obtain care. This is worrying because there were very few public and private hospitals within the local government area and scattered all over the place. This suggests that seeking or deploying MCH services at secondary care may not be cost effective. These public and private hospitals are not within easy reach to the women than health centers and posts, and should constitute referral centers [secondary care].

In the study area, free maternal and child health services were offered at the primary health care centers as the first point of call. The findings from this study indicate that mothers may not utilize these services at the PHC level, and perhaps bypass the formal treatment seeking algorithm. More so, resources may be wasting at the primary care level since the women do not know that these services exist although this study did not evaluate benefit incidence analysis [BIA] of the free services at primary level.

Nevertheless, the findings show that women indicated that MCH services were available at pharmacies, patent medicine dealer’s shops and TBAs. These findings are therefore quite challenging for reducing child mortality and improving maternal health in southeast Nigeria. By policy, MCH targets to increase skilled births thereby reducing maternal and child deaths, and in Nigeria, these services are offered by recognized public and private hospitals, and primary health centers. Such services as ANC, immunization, growth monitoring, breastfeeding initiative, supplementary feeding, delivery, family planning and oral rehydration therapy are never offered outside the formal health facilities [i.e. recognized public and private hospitals, and primary health centers]. This implies that if by chance pregnant women go to these places to seek care, they would be patronizing quacks. In addition, this places TBAs and patent medicine dealers shops are wide spread in the study area, and closer to where people live. Therefore interventions to scale up delivery of MCH services in the area should target both the consumers and the providers especially the TBAs, pharmacies and patent medicine dealers to ensure that they know and practice appropriate referral services if consumers should consult them.

## Conclusion

Knowledge of available services is important for consumers to make use of the services. Awareness programmes should be targeted more on the consumers if the MDG 4 and 5 must be reached by 2015.

## Competing interests

The authors declare that they have no competing interest.

## Authors’ contributions

NEK developed the draft, conducted data collection and analysis, EGN developed the protocol for the study, and supervised data collection, UCU contributed in producing the draft. All authors read through the manuscripts and responded to all the reviewers comments. All authors read and approved the final manuscript.

## Pre-publication history

The pre-publication history for this paper can be accessed here:

http://www.biomedcentral.com/1472-6963/13/53/prepub

## Supplementary Material

Additional file 1 Questionaire. Click here for file
