# Supplementary material for: Consumer knowledge and availability of maternal and child health services: a challenge for achieving MDG 4 and 5 in Southeast Nigeria
Source: BMC Health Serv Res. 2013 Feb 9;13:53. doi: 10.1186/1472-6963-13-53 (PMC3607887; doi:10.1186/1472-6963-13-53)
Supplement: Additional file 1 — Questionaire. [file 1472-6963-13-53-S1.docx]

**QUESTIONAIRE**

Dear respondent, the bearer is carrying out a research on the determinants of utilization of maternal and child health services in public health facilities in Ezeagu Local Government Area. Your cooperation is highly appreciated.

**Instruction:**

write **(1)** if your Answer is yes and **(0)** If your answer is No in the columns provided.

**Section 1** : **Demographic and socio – economic data**

**1.** **Age:**

(1a) 16 -25yrs [ ] (1b) 26- 35yrs [ ]

(1c) 36-45yrs [ ] (1d) 46-55yrs [ ]

(1e) > 55yrs [ ]

**2**. **Marital status**:

(2a) Single [ ] (2b) Married [ ]

(2c) Widowed [ ] (2d) Divorced [ ]

(2e) Separated [ ]

**3.** No of children (specify) _________

**4.** **Educational Status**:

(4a) No formal education [ ] (4b) First school leaving [ ]

(4c) WASC/NECO [ ] (4d) Diploma [ ]

(4e) NCE [ ] (4g) BSC [ ]

(4h) MSC [ ] (4i) PhD [ ]

**5. Occupation**

(5a) Civil servant [ ] (5b) Trading [ ]

(5c) Farming [ ] (5d) Un-employed [ ]

(5e) Professional [ ]

**6. Religion**

(6a) Traditional [ ] (6b) Christianity [ ] (6c) Islam [ ]

**Section 2: Knowledge about MCH services**

**7. What maternal health services are available in your community?**

(7a) Antenatal care [ ] (7b) Delivery services [ ]

(7c) Post natal services [ ] (7d) Family planning services [ ]

(7e) Health education [ ] (7f) Others [ ]

**8 Where are the Maternal health Services available in your community?**

(8a) Public hospitals [ ] (8b) Private hospitals [ ]

(8c) PHC centres [ ] (8d) Pharmacy [ ]

(8e) Patent medicine shop [ ] (8f) TBA [ ]

**9. What child health services are available in your community?**

(9a) Oral rehydration therapy [ ] (9b) Immunization services [ ]

(9c) Growth monitoring services [ ] (9d) Breast feeding Initiatives [ ]

(9e) Others [ ]

**10 Where are the child health services available in your community?**

(10a) Public hospitals [ ] (10b) Private hospitals [ ]

(10c) PHC centres [ ] (10d) Pharmacy [ ]

(10e) Patent medicine shop [ ] (10f) TBA [ ]

**Section 3: Utilization of MCH Services in your community**

**11. What factors influence your use of both maternal and child health services in your community?**

(11a) Influence of family members [ ] (11b) Mothers education [ ]

(11c) Traditional practices/culture [ ] (11d) Availability of alternative care [ ]

(11e) Proximity to the health facility [ ] (11f) Inability to pay for health care [ ]

(11g) Availability of qualified health

personnel [ ]

**Section 4: Experience and practice of MCH Services**

**12. How long have you used: Maternal health services**

(12a) 3-6years [ ] (12b) 7-12years [ ]

(12c) 13-18years [ ] (12d) 19-24years [ ]

(12e) > 24years [ ]

**13. How long have you used: child health services ?**

(13a) 3-6years [ ] (13b) 7-12years [ ]

(13c) 13-18years [ ] (13d) 19-24years [ ]

(13e) > 24years [ ]

**14. What are the problems encountered with the utilization of MCH services ?**

(14a) Affordability [ ] (14b) Accessibility [ ]

(14c) Availability [ ] (14d) Acceptability [ ]

**Section 5: Recommendations for Improving MCH services in your**

**Community**

**15. What do you think can be done to improve maternal health services in your community?**

(15a) Reducing waiting time [ ] (15b) Subsidizing health bills [ ]

(15c) Obtaining informed opinion of

the community [ ] (15d) Giving constant health Information [ ]

(15e) Improving the provision of

alternative care [ ] (15f) Removing user fees [ ]

**16. What do you think can be done to improve child health services in your community?**

(15a) Reducing waiting time [ ] (15b) Subsidizing health bills [ ]

(15c) Obtaining informed opinion of

the community [ ] (15d) Giving constant health Information [ ]

(15e) Improving the provision of

alternative care [ ] (15f) Removing user fees [ ]
